# Unveiling the Causal Roles of the Oral Microbiome-Host Metabolism-Inflammation Axis in Periodontitis: A Mendelian Randomisation Study

**DOI:** 10.3290/j.ohpd.c_2284

**Published:** 2025-09-26

**Authors:** Ziyu Zhao, Weihong Yang, Yingying Hu

**Affiliations:** a Ziyu Zhao Department of Stomatology, The Third Hospital of Changsha (The Affiliated Changsha Hospital of Hunan University), Tianxin District, Changsha, China; Hunan University of Chinese Medicine, Yuelu District, Changsha, China. Data analysis, wrote the paper.; b Weihong Yang Changsha Stomatological Hospital, Tianxin District, Changsha, China. Data analysis, wrote the paper.; c Yingying Hu Department of Stomatology, The Third Hospital of Changsha (The Affiliated Changsha Hospital of Hunan University), Tianxin District, Changsha, China. Study design and conception, data analysis, wrote the paper.

**Keywords:** periodontitis, oral microbiota, blood metabolome, inflammatory proteins, Mendelian randomisation

## Abstract

**Purpose:**

Periodontitis is a common chronic inflammatory disease with a complex pathogenesis involving oral microbiota dysbiosis, systemic metabolic disturbances, and inflammatory responses. Although observational studies have suggested close associations between these biological factors and periodontitis, the causal relationships remain unclear. Elucidating the causal roles and interactions of these factors is crucial for effective prevention and treatment of the disease.

**Methods:**

In this study, we utilised large-scale genome-wide association study data and applied a two-sample Mendelian randomisation (MR) framework. We systematically evaluated the independent causal effects of 43 oral microbial taxa, 1,400 blood metabolites, and 91 inflammatory proteins on periodontitis risk. Direct effects were assessed using univariable MR (UVMR), while multivariable MR (MVMR) was used to analyse interactions. Mediation MR analysis was further performed to explore potential causal pathways.

**Results:**

UVMR analysis found that family *Pasteurellaceae* (OR = 0.953) and genus *Veillonella* (OR = 0.943) were statistically significantly associated with a reduced risk of periodontitis. Further analyses revealed 45 blood metabolites and 4 inflammatory proteins with genetically supported causal relationships to periodontitis. Mediation analysis indicated that the protective effect of family *Pasteurellaceae* on periodontitis risk is partially mediated by the regulation of circulating 4-hydroxychlorothalonil, with a mediation proportion of 3.54%.

**Conclusion:**

This study provides the first systematic genetic evidence for the causal roles and potential mediation mechanisms of oral microbiota, blood metabolites, and inflammatory proteins in the development of periodontitis. The findings offer new insights into the role of the oral microbiome-host metabolism-inflammation axis in periodontitis aetiology.

Periodontitis is a chronic inflammatory disease triggered by persistent stimulation from subgingival microbial biofilms. It leads to irreversible destruction of periodontal supporting tissues and ultimately results in tooth loss.^21^ This disease is highly prevalent, affecting approximately 20–50% of the global population, and thus represents a significant public health burden.^24^ As there is currently no effective cure for periodontitis, preventive strategies targeting its aetiology are crucial. The pathogenesis of periodontitis is recognised as multifactorial, involving dysbiosis of the oral microbiota, host susceptibility-mediated inflammatory responses, environmental risk factors, and underlying systemic metabolic disturbances.^11,15^ Although these associations are increasingly understood, the complex causal pathways mediating their effects and their specific contributions to periodontitis risk remain to be fully elucidated.^29^ This limitation constrains our understanding of the disease’s underlying mechanisms and the development of innovative interventions. Additionally, a clear understanding of the microbial aetiology, placed within contemporary perspectives, is fundamental to identifying critical pathogenic factors, discovering novel preventive targets, and optimising therapeutic strategies.^3^ Therefore, investigating the causal effects of the oral microbiota, blood metabolites, and inflammatory proteins in the onset and progression of periodontitis is of key scientific importance for identifying critical pathogenic factors, discovering novel preventive targets, and optimising therapeutic strategies.

Numerous observational studies have suggested that oral microbiota dysbiosis, systemic metabolic alterations, and changes in inflammatory proteins are closely associated with periodontitis development.^15^ For example, enrichment of specific microbes such as Porphyromonas gingivalis and Tannerella forsythia is widely recognised as related to periodontitis, while changes in the relative abundance of other taxa, including genus *Veillonella* and family *Pasteurellaceae*, have also been observed in relation to periodontitis.^9,13^ Similarly, metabolomics studies have revealed significant alterations in endogenous small-molecule metabolites in blood, saliva, and gingival crevicular fluid from periodontitis patients, involving pathways such as amino acid metabolism, lipid dysregulation, and oxidative stress.^2^ Given the central role of inflammation in periodontitis, various pro-inflammatory cytokines, including IL-1β, IL-6, TNF-α, IL-10, and other inflammation-related proteins, have been reported to be dysregulated in patients, reflecting both local and systemic inflammatory disturbances.^44^ However, while observational studies provide important clues regarding potential risk factors, their inherent limitations in inferring causality – such as susceptibility to confounding and reverse causation – cannot be overlooked.^22^ Consequently, a central imperative in contemporary periodontology is to transcend conventional diagnostics, which predominantly capture the historical sequelae of cumulative tissue destruction rather than the incipient pathological processes driving the disease.^20^ The search for reliable biomarkers, particularly microbial signatures that can signal a dysbiotic state before irreversible damage occurs, is a major focus, though translating these into clinically applicable tools remains a significant challenge.^31^ To bridge this critical gap between association and causation and to provide a mechanistic foundation for predictive diagnostics, methods capable of robustly inferring causality are urgently needed.

To address this critical need, Mendelian randomisation (MR) analysis uses genetic variants as instrumental variables (IVs) for exposures, simulating the design principles of randomised controlled trials. This approach can effectively avoid confounding and reverse causation common in traditional observational studies, thus providing more robust evidence for causal relationships between exposures and outcomes.^46^ In recent years, MR has been successfully applied to explore potential causal links between the oral microbiome and various complex diseases, such as certain cancers and type 2 diabetes, identifying several oral bacterial taxa that may play roles in disease pathophysiology.^12^ Similarly, MR-based blood metabolomics studies have revealed potential causal relationships between circulating metabolites and inflammatory diseases, metabolic syndrome, and other complex traits, contributing new insights into disease metabolism.^47^ MR analysis has also been used to assess the causal effects of inflammatory proteins in immune-mediated diseases, cancers, and neuropsychiatric disorders, providing genetic support for the role of inflammation in these conditions.^4^ Although previous studies have examined these factors individually in relation to periodontitis, there is a lack of systematic mediation MR studies integrating oral microbiome, blood metabolome, and inflammatory proteome data to comprehensively assess their independent and potentially mediated causal effects on periodontitis risk. In particular, the complex causal chains and mediation pathways among these factors remain to be clarified.

Given the existing knowledge gaps, we formulated a central hypothesis: some components of the oral microbiota causally influence periodontitis risk, and this effect is at least partially mediated through systemic host metabolic and inflammatory pathways. To test this hypothesis, our study has three primary aims. First, we aim to systematically dissect the independent causal effects of oral microbiota, circulating blood metabolites, and inflammatory proteins on the risk of periodontitis using a comprehensive MR framework. Second, we aim to identify potential causal chains by investigating whether periodontitis-associated microbes also causally influence specific metabolites or proteins. Finally, through multivariable and mediation MR, we aim to elucidate and quantify these potential mediating pathways, thereby providing genetic evidence for the existence of an ‘oral microbiome-host metabolism-inflammation’ axis in periodontitis aetiology. By applying this integrative, multi-omics causal inference approach, we aim to deepen our understanding of periodontitis aetiology and identify key biomolecules and pathways associated with disease risk. These insights provide a scientific basis for novel preventive and therapeutic strategies, as well as for improved risk assessment and early warning.

## METHODS

### Overall Framework and Analytical Workflow

**Fig 1:**
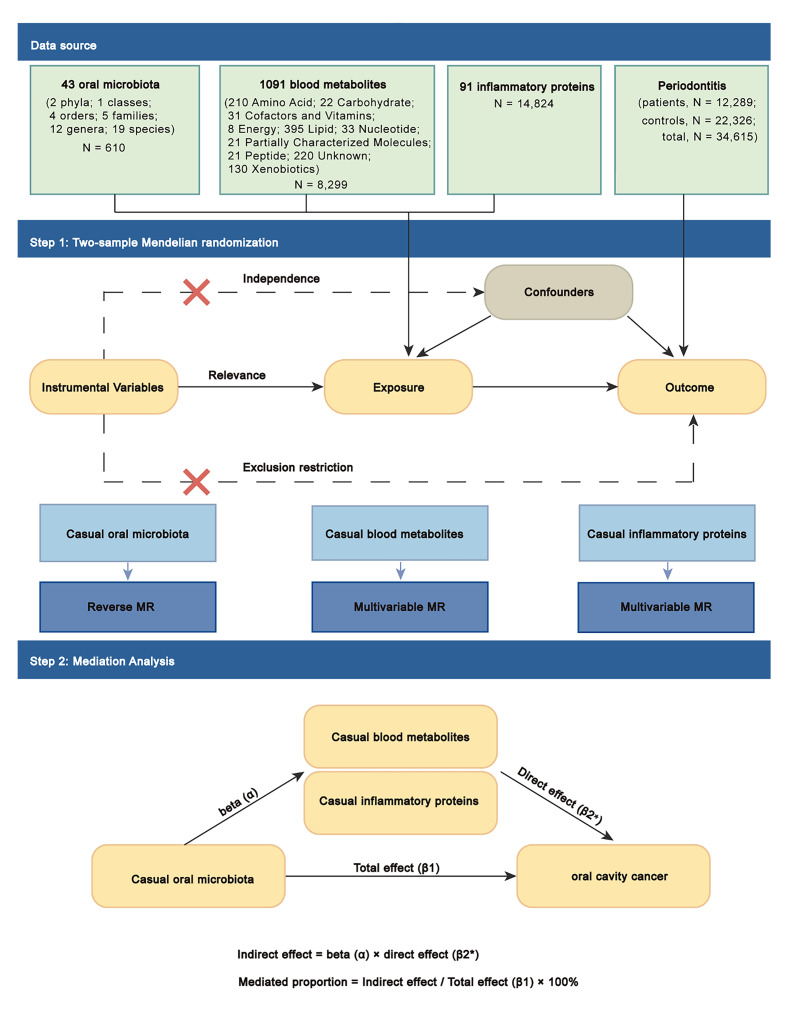
Study design for Mendelian randomisation analysis of oral microbiota, blood metabolites, inflammatory proteins, and periodontitis. This figure illustrates the two-sample MR and mediation MR framework. Analysis steps: (1) Two-sample MR to assess causality; (2) Mediation MR to evaluate blood metabolites and inflammatory proteins as mediators. Methods include inverse-variance weighted (IVW), MR-Egger, and weighted median approaches.

To investigate the complex roles of the oral microbiota in the aetiology and progression of periodontitis, and to clarify the potential mediating roles of blood metabolites or inflammatory proteins, we constructed a systematic MR analytical framework based on genome-wide association studies (GWAS) summary data. Figure 1 delineates this analytical workflow. The workflow began with univariable MR (UVMR) to assess the direct causal effects of the oral microbiota on periodontitis, using relevant GWAS summary statistics (see ‘Data Sources and Description’ section) and rigorously selected genetic IVs (see ‘Selection and Validation of Genetic Instrumental Variables’). Reverse UVMR was also performed to explore the potential impact of periodontitis on the oral microbiota. Next, similar UVMR analyses were conducted with blood metabolites or inflammatory proteins as outcomes, to identify circulating factors significantly influenced by the oral microbiota. Subsequently, UVMR was applied again to test whether these microbiota-driven factors themselves have causal effects on periodontitis risk, thereby identifying potential mediators that meet both criteria (see ‘Univariable Mendelian Randomisation Analysis’). Finally, multivariable MR (MVMR) analysis (see ‘Multivariable Mendelian Randomisation Analysis’) was used to precisely elucidate and quantify the roles of these potential mediators. To ensure the robustness and validity of all MR results, a series of systematic sensitivity analyses were performed (see ‘Heterogeneity, Pleiotropy, and Sensitivity Analysis’), rigorously testing the core MR assumptions and enhancing the reliability of the conclusions. This study follows the STROBE-MR reporting guidelines, as detailed in Supplementary Table S1 41.

### Mendelian Randomisation Principles and Core Assumptions

Traditional observational epidemiology is often confounded by unmeasured factors and reverse causation when exploring associations such as those between the oral microbiota and periodontitis. MR analysis uses genetic variants as IVs, leveraging the random allocation of genotypes during meiosis to overcome these limitations and obtain potentially unbiased estimates of causal effects, provided certain core assumptions are met. The validity of MR analysis depends on three key assumptions: (1) the relevance assumption, requiring that IVs are robustly associated with the exposure; (2) the independence assumption, stipulating that IVs affect the outcome only through the exposure; and (3) the exclusion restriction assumption, requiring that IVs are not associated with any confounders of the exposure-outcome relationship. Our study design explicitly considered and carefully tested these assumptions to maximise the validity and reliability of the MR results.^25^

### Data Sources and Description

We utilised four publicly available large-scale GWAS summary datasets covering the exposures, potential mediators, and outcome of interest. Oral microbiome data were derived from a study of 610 Danish participants,^42^ providing statistics for 43 distinct oral microbial taxa. Periodontitis GWAS data came from a study involving 12,289 cases and 22,326 controls.^40^ Blood metabolite summary statistics were obtained from the Canadian Longitudinal Study on Aging,^10^ which included 51,338 individuals and 1,400 metabolite categories, such as amino acids, carbohydrates, lipids, and nucleotides. Finally, GWAS data for 91 inflammation-related proteins were sourced from a study of 14,824 individuals of European ancestry.^49^ The participants in these studies were predominantly of European descent, with minimal expected sample overlap, supporting the MR assumption of sample independence.

### Selection and Validation of Genetic Instrumental Variables

To ensure the validity and reliability of MR analysis, we followed strict criteria for selecting and validating genetic IVs for exposures. First, considering potential sample size limitations, we used a P-value threshold of <5 × 10^-^⁵ for initial single-nucleotide polymorphism (SNP) selection, balancing instrument strength with the need for sufficient IVs to maintain statistical power.^6^ Second, to minimise linkage disequilibrium (LD) among IVs, we performed LD clumping (kb = 10,000, r^2^ <0.001) based on the 1000 Genomes Project Phase 3 European reference panel, ensuring approximate independence of selected IVs. Third, to reduce weak instrument bias, we calculated the F-statistic for each SNP (F = (β/SE)^2^), retaining only those with F ≥ 10 as strong IVs for subsequent MR analysis.^34^

### Univariable Mendelian Randomisation Analysis

In the first step of our design, we performed UVMR to estimate the causal effect of each exposure on each outcome using the relevant genetic IVs. To address potential bias from horizontal pleiotropy, we employed three complementary MR methods from the ‘TwoSampleMR’ R package. The inverse-variance weighted (IVW) method^14^ served as the primary analysis, providing the most precise estimates under the assumption of no or balanced pleiotropy. To enhance robustness against invalid instruments, we used the weighted median method,^5^ which can yield reliable estimates even if up to 50% of IVs are pleiotropic. Additionally, MR-Egger regression^35^ was applied to detect directional pleiotropy and provide potentially corrected causal estimates, though with lower statistical power. Comparing results across these methods helps assess the robustness of causal estimates. Metabolites or proteins showing significant causal associations were identified as potential mediators for subsequent MVMR analysis.

### Multivariable Mendelian Randomisation Analysis

To clarify and validate the roles of potential mediators identified by UVMR in the overall causal pathway from the oral microbiota to periodontitis, we applied MVMR.^16^ As an extension of UVMR, MVMR allows simultaneous estimation of the direct causal effects of multiple exposures (a microbial taxon and a potential mediator) on the outcome (periodontitis), while adjusting for genetic correlations among exposures. Its key advantage is the ability to distinguish the direct effect of the oral microbiota (independent of the mediator) from the effect of the mediator (adjusted for the microbiota). Using the product of coefficients method,^30^ we quantified the indirect effect by multiplying the UVMR estimate for the microbiota-mediator effect (β1) by the MVMR estimate for the mediator-periodontitis effect (β2). The mediation proportion was then calculated as (β1 × β2)/β3, where β3 is the total microbiota-periodontitis effect from UVMR. Thus, MVMR provides deeper causal inference regarding potential mechanistic pathways and the importance of identified mediators.

### Heterogeneity, Pleiotropy, and Sensitivity Analysis

To rigorously assess the reliability of MR results and test core assumptions, we conducted a comprehensive set of sensitivity analyses. First, Cochran’s Q statistic was used to evaluate heterogeneity among IVs (P <0.05). Second, two complementary methods were used to assess potential horizontal pleiotropy: the MR-Egger intercept test (P < 0.05) and the MR-PRESSO test. MR-PRESSO can identify and help remove pleiotropic outlier IVs, providing corrected estimates after outlier removal (P <0.05). If MR-PRESSO detected outliers, the MR analysis was repeated after their exclusion. These systematic analyses collectively provide multiple safeguards for the validity of MR results and enhance the credibility of the study’s conclusions.

### RESULTS

### Causal Effects of Oral Microbiota on Periodontitis

**Fig 2:**
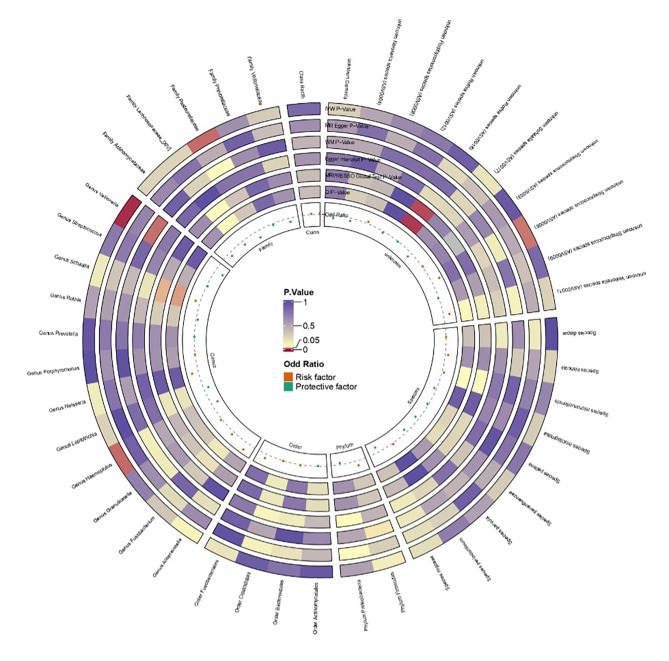
Causal associations between oral microbiota and periodontitis using two-sample Mendelian randomisation. Odds ratios (OR) and 95% confidence intervals (95% CI) were estimated using the inverse-variance weighted (IVW) method, with sensitivity analyses via MR-Egger and weighted median methods. Significance was determined at P <0.05.

To assess whether oral microbiota dysbiosis is a potential etiological factor for periodontitis, we first used UVMR to test causal associations between multiple oral microbial taxa and periodontitis risk. The analysis identified two taxa – (OR = 0.953, 95% CI: 0.916–0.992, P = 0.017) and genus *Veillonella* (OR = 0.943, 95% CI: 0.905–0.982, P = 0.005) – with statistically significant protective effects against periodontitis (Fig 2; Supplementary Table S2). Results from the weighted median and MR-Egger methods were consistent with the primary IVW analysis (Supplementary Table S2). To evaluate potential reverse causality, we conducted reverse MR analysis (Supplementary Table S3). No causal effect of periodontitis on family *Pasteurellaceae* abundance was found. However, for genus *Veillonella*, reverse MR revealed a statistically significant causal effect of periodontitis in increasing its abundance (OR = 2.642, 95% CI: 1.352–5.163, P = 0.004). This suggests that periodontitis itself can influence Veillonella abundance, rather than being solely affected by this microbe. MR-PRESSO detected no statistically significant horizontal pleiotropy due to outlier SNPs (P >0.05), and the MR-Egger intercept also showed no evidence of directional pleiotropy (P >0.05) (Supplementary Table S4). Overall, the protective causal association between family *Pasteurellaceae* and periodontitis risk appears robust. For genus *Veillonella*, although a protective effect was identified, reverse MR indicates that periodontitis may also causally increase its abundance.

### Causal Effects of Blood Metabolites on Periodontitis

**Fig 3:**
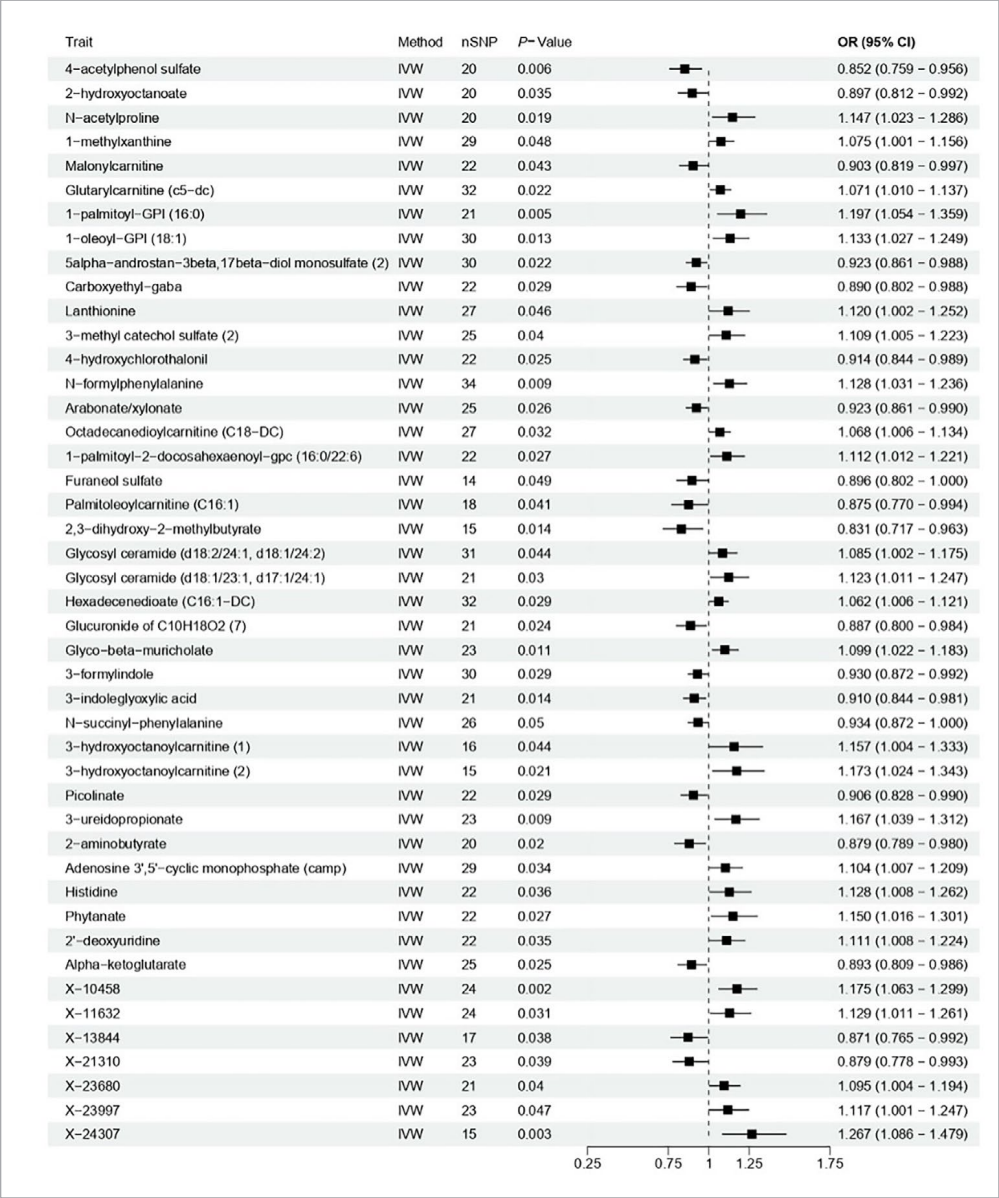
Causal associations between blood metabolites and periodontitis using two-sample Mendelian randomisation. The inverse-variance weighted method was primary, supported by MR-Egger and weighted median methods. Significance was determined at P <0.05.

Given the key role of metabolic dysregulation in inflammatory diseases, we further evaluated the causal relationships between numerous blood metabolites and periodontitis risk. The IVW method identified 45 blood metabolites with statistically significant causal associations with periodontitis (Fig 3; Supplementary Table S5). These metabolites span various biochemical categories, including acylcarnitines, sulfated compounds, and lysophospholipids, suggesting that widespread systemic metabolic alterations may be involved in periodontitis pathogenesis. Among these, several metabolites showed particularly strong associations. For example, 1-palmitoyl-GPI (16:0) (OR = 1.197, 95% CI: 1.054–1.359, P = 0.005), N-formylphenylalanine (OR = 1.128, 95% CI: 1.031–1.236, P = 0.009), and 3-ureidopropionate (OR = 1.167, 95% CI: 1.039–1.312, P = 0.009) were associated with increased risk, while 4-acetylphenol sulfate (OR = 0.852, 95% CI: 0.759–0.956, P = 0.006), 2,3-dihydroxy-2-methylbutyrate (OR = 0.831, 95% CI: 0.717–0.963, P = 0.014), and 3-indoleglyoxylic acid (OR = 0.910, 95% CI: 0.844–0.981, P = 0.014) showed protective effects (Fig 3; Supplementary Table S5). The robustness of these findings was largely confirmed by the weighted median and MR-Egger methods (Supplementary Table S5). Importantly, the MR-Egger intercept showed no evidence of horizontal pleiotropy (P >0.05), and the MR-PRESSO global test did not identify influential outlier SNPs (P >0.05) (Supplementary Table S6). The consistency of results across methods strongly suggests that the identified causal associations between these 45 blood metabolites and periodontitis risk are robust, highlighting the potential importance of systemic metabolic changes in periodontitis aetiology and pointing to these metabolites as candidate biomarkers or therapeutic targets.

### Causal Effects of Inflammatory Proteins on Periodontitis

**Fig 4:**
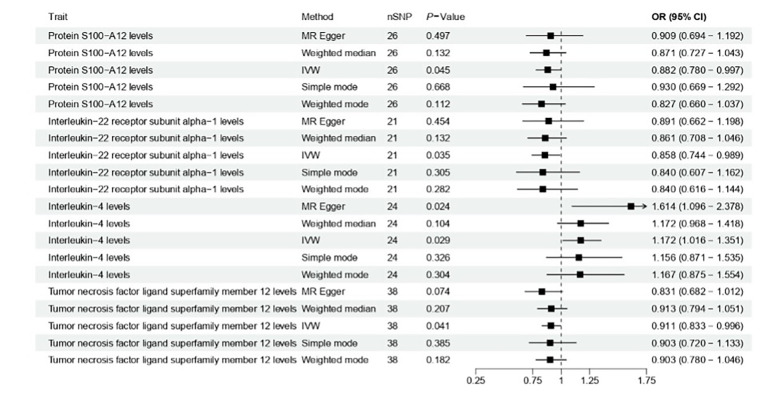
Causal associations between inflammatory proteins and periodontitis using two-sample Mendelian randomisation. The inverse-variance weighted method was used, with MR-Egger and weighted median validation. Significance was determined at P <0.05.

Given the established role of inflammation in periodontitis, we further explored potential causal associations between inflammatory proteins and periodontitis risk. Among the 91 proteins evaluated, MR analysis identified four with statistically significant causal relationships (Fig 4; Supplementary Table S7). Genetically predicted higher levels of interleukin-4 (IL-4) were associated with increased periodontitis risk (OR = 1.172, 95% CI: 1.016–1.351, P = 0.029). In contrast, higher levels of interleukin-22 receptor subunit alpha-1 (IL-22RA1) (OR = 0.858, 95% CI: 0.744–0.989, P = 0.035), S100 calcium-binding protein A12 (S100-A12) (OR = 0.882, 95% CI: 0.780–0.997, P = 0.045), and tumour necrosis factor ligand superfamily member 12 (TNFSF12) (OR = 0.911, 95% CI: 0.833–0.996, P = 0.041) were associated with reduced risk. Sensitivity analyses and MR-Egger regression supported the reliability of these associations (P >0.05) (Supplementary Table S8).

### Mediation Analysis Results

**Fig 5:**
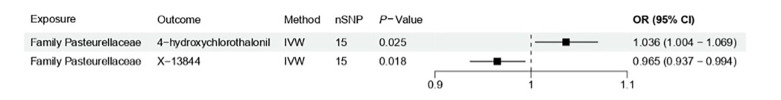
Causal associations between oral microbiota and blood metabolites using two-sample Mendelian randomisation. The inverse-variance weighted method was used, with MR-Egger and weighted median validation. Significance was determined at P <0.05.

Building on the established independent causal associations of the oral microbiota, blood metabolites, and inflammatory proteins with periodontitis, we performed two-step MR analyses. First, UVMR was used to assess the potential causal effects of the two periodontitis-associated oral microbial taxa on related blood metabolites or inflammatory proteins (Fig 5; Supplementary Table S9). Specifically, family *Pasteurellaceae* showed a significant positive association with 4-hydroxychlorothalonil (OR = 1.036, 95% CI: 1.004–1.069, P = 0.025) and a significant negative association with X-13844 (OR = 0.965, 95% CI: 0.937–0.994, P = 0.018). Considering the directionality, 4-hydroxychlorothalonil was identified as a potential mediator. No inflammatory proteins were identified as mediators. Subsequently, MVMR analysis was conducted to assess whether the observed association with 4-hydroxychlorothalonil mediated the effect of family *Pasteurellaceae* on periodontitis risk. The MVMR results showed that, after accounting for family *Pasteurellaceae*, the protective association of 4-hydroxychlorothalonil with periodontitis remained significant (OR = 0.954, 95% CI: 0.919–0.990, P = 0.012) (Table 1), with a mediation proportion of 3.54%, representing a modest but detectable contribution. These results suggest a preliminary pathway where family *Pasteurellaceae* may exert its protective effect on periodontitis risk partly by influencing circulating levels of 4-hydroxychlorothalonil.

**Table 1:** Mediation analysis of oral microbiota effects on periodontitis via blood metabolites

Exposure	Adjusted factors	Inverse variance weighted
OR	95%CI	P value	Mediation proportion
4-hydroxychlorothalonil	Family *Pasteurellaceae*	0.954	0.919–0.990	0.012	3.54%
Family *Pasteurellaceae*	4-hydroxychlorothalonil	0.944	0.868–1.027	0.182	


## DISCUSSION

Using a two-sample MR framework, this study systematically evaluated the potential causal relationships between the oral microbiota, blood metabolites, and inflammatory proteins and periodontitis risk, and further explored possible mediation pathways among them. The results provided genetic evidence for significant associations between genetically predicted levels of family *Pasteurellaceae*, 45 blood metabolites, and 4 inflammatory proteins (IL-4, IL-22RA1, S100-A12, TNFSF12) and periodontitis susceptibility. Notably, our mediation analysis identified a preliminary pathway, suggesting that family *Pasteurellaceae* may exert a protective effect on periodontitis risk by modulating circulating levels of 4-hydroxychlorothalonil. These findings provide new genetic evidence for the complex aetiology of periodontitis and offer important clues for developing novel biomarkers, preventive strategies, and therapeutic targets.

We observed a statistically significant protective causal relationship between increased abundance of family *Pasteurellaceae* and reduced periodontitis risk. This finding contrasts with some traditional views, which associate certain members of this family, such as *Aggregatibacter actinomycetemcomitans*, with periodontitis development.^23^ The difference may arise because MR reflects the cumulative effect of genetically driven long-term exposure, rather than a snapshot of microbial composition during disease states. Alternatively, at the family level, the protective effects of commensal members may outweigh the negative impact of specific pathogens.^18^ Similarly, our study found that increased relative abundance of genus *Veillonella* may have a protective effect against periodontitis, consistent with cross-sectional studies showing higher *Veillonella* abundance in healthy individuals.^7^ Importantly, reverse MR analysis indicated that periodontitis itself may causally increase *Veillonella* abundance, suggesting a complex reciprocal interaction. It implies *Veillonella* may function as both a protective agent that mitigates disease and a sensitive biomarker whose proliferation is a consequence of the host’s inflammatory environment.^8,33^ In summary, our MR analysis highlights the multidimensional roles of family *Pasteurellaceae* and genus *Veillonella* in periodontitis, warranting further investigation into their biological mechanisms and clinical significance.

Given the central role of metabolic dysregulation in inflammatory diseases, we further evaluated the causal associations between blood metabolites and periodontitis risk. Our results showed that genetically predicted levels of multiple blood metabolites are statistically significantly associated with periodontitis susceptibility, suggesting that widespread systemic metabolic changes may contribute to disease pathogenesis.^39^ For example, 1-palmitoyl-GPI (16:0), a lysophosphatidylinositol (LPI), was associated with increased risk. LPIs are bioactive lipids known to activate downstream signalling pathways via receptors such as GPR55, regulating inflammation, cell proliferation, and migration.^1^ Given that periodontitis is characterised by chronic inflammation and tissue destruction, elevated genetically predicted 1-palmitoyl-GPI (16:0) may increase susceptibility by enhancing pro-inflammatory responses or promoting pathological cellular activities. N-formylphenylalanine, a potential bacterial or mitochondrial formyl peptide chemoattractant, was also associated with increased risk, possibly due to its ability to recruit neutrophils and exacerbate local inflammation via formyl peptide receptors.^26^ 3-ureidopropionate, an intermediate in pyrimidine catabolism, was linked to increased risk, potentially reflecting nucleic acid metabolic disturbances or increased cell damage, which are associated with inflammation; related metabolites such as β-alanine have been implicated in neuroinflammation and pain sensitisation.^45^ Conversely, higher genetically predicted levels of 3-indoleglyoxylic acid were associated with reduced risk. Microbiota-derived tryptophan metabolites, especially indole derivatives, are known to modulate host immunity and inflammation, often via activation of the aryl hydrocarbon receptor pathway, which regulates immune cell function and maintains mucosal barrier integrity.^38,43^ Thus, higher 3-indoleglyoxylic acid may indicate a beneficial microbial tryptophan metabolism profile, producing immunoregulatory and anti-inflammatory compounds that help mitigate systemic inflammation and protect against periodontitis. Overall, our MR analysis provides genetic evidence for causal relationships between a range of blood metabolites and periodontitis risk, supporting the importance of systemic metabolic changes in disease aetiology and offering valuable leads for biomarker discovery and therapeutic intervention, though further research is needed to clarify their biological functions and clinical applications.

Recognising the central role of inflammation in periodontitis progression, we also explored the causal relationships between inflammatory proteins and periodontitis risk. Our results identified statistically significant associations for IL-4, IL-22RA1, S100-A12, and TNFSF12. Specifically, genetically predicted higher IL-4 levels were associated with increased risk. IL-4 is a classic Th2 cytokine, traditionally involved in antibody responses and allergic inflammation, and has shown both anti-inflammatory and bone-protective effects in some models of periodontitis.^27^ Although IL-4 is generally thought to promote M2 macrophage polarisation and suppress Th1 responses, its role in chronic inflammatory diseases like periodontitis may be more complex.^48^ Persistent or dysregulated IL-4 signalling may lead to immune suppression, impaired pathogen clearance, or promote fibrosis, potentially explaining the observed risk effect.^32^ In contrast, higher genetically predicted levels of IL-22RA1, S100-A12, and TNFSF12 were associated with reduced risk. IL-22RA1, as a receptor for IL-22, is known for its role in mucosal barrier protection and epithelial repair, suggesting that increased IL-22RA1 may enhance periodontal tissue resilience.^17^ Although S100-A12 and TNFSF12 are often reported as upregulated in periodontitis and involved in pro-inflammatory processes,^19,28^ they may also have regulatory or protective roles under certain conditions. These findings underscore the importance of host immune genetic susceptibility in periodontitis pathogenesis, providing new perspectives for understanding immune pathology and suggesting these proteins as potential biomarkers or therapeutic targets. However, given the pleiotropy and complex regulation of these cytokines and proteins, further experimental studies are needed to clarify their precise roles and interactions in periodontitis.

Through MVMR analysis, we also found that the previously identified protective effect of family *Pasteurellaceae* on periodontitis risk may be partly mediated by its influence on circulating 4-hydroxychlorothalonil. This finding aligns with the broader recognition that the microbiota, including oral and gut microbes, can affect host physiology and pathology via their metabolites,^37^ suggesting that oral microbes may modulate periodontitis risk by altering specific systemic metabolite profiles. 4-hydroxychlorothalonil is a known metabolite of the fungicide chlorothalonil, but its endogenous protective function in humans is not fully understood. The microbiota, including oral microbes, are known to participate in the metabolism of xenobiotics, altering the bioactivity and host effects of these compounds and their metabolites.^36^ Thus, family *Pasteurellaceae* may influence the transformation and generation of 4-hydroxychlorothalonil through its metabolic capacity, thereby regulating its circulating levels. If 4-hydroxychlorothalonil possesses unknown anti-inflammatory or immunomodulatory properties, or if its formation is accompanied by other beneficial metabolic byproducts, this could explain its observed mediating protective effect. While our findings provide new causal evidence for how oral microbes may influence periodontitis risk via host metabolic changes, these results are preliminary. The precise biological functions, main sources, and mechanistic contributions of specific mediating metabolites in periodontitis pathophysiology remain important topics for future research.

The causal insights from this study offer a translational roadmap for periodontitis management. The identified metabolites and proteins represent high-priority candidates for novel biomarkers aimed at early risk assessment, potentially enabling targeted preventive care long before clinical signs appear. Furthermore, our findings provide a genetic rationale for new therapeutic interventions, including host-modulation therapies targeting specific metabolic pathways (eg, LPI signalling, tryptophan metabolism) and immunomodulatory drugs focused on cytokine axes, such as IL-4 and IL-22. Finally, the preliminary mediation pathway involving family *Pasteurellaceae* hints at the potential for microbiome-based strategies that modulate systemic metabolism to prevent periodontitis. Functional validation of these targets and pathways is now a critical next step toward translating these genetic discoveries into clinical practice. Several limitations of this study should be considered. First, most GWAS summary data used were from individuals of European ancestry, which may limit the generalisability of findings to other populations. Second, although we used multiple sensitivity analyses to assess and correct for potential horizontal pleiotropy, we cannot completely rule out the possibility that some genetic IVs may influence periodontitis risk through biological pathways independent of the studied exposures. Specifically, the mediation finding for 4-hydroxychlorothalonil could be confounded by pleiotropy if the IVs for *Pasteurellaceae* also influence the host’s capacity to metabolise the parent environmental compound. Furthermore, our use of a relaxed significance threshold (P <5 × 10-5) for selecting IVs, while necessary to ensure an adequate number of IVs for the analysis, may increase the potential for weak instrument bias. Additionally, the strength and coverage of available genetic IVs for some exposures may be limited, potentially affecting the precision and power of causal estimates. Future studies should aim to validate these findings in more diverse cohorts and integrate multi-omics data and functional experiments to clarify the molecular mechanisms and complex interaction networks of identified microbes, metabolites, and proteins in periodontitis. Further confirmation and mechanistic analysis of key nodes in mediation pathways, as well as in-depth evaluation of biomarkers and therapeutic targets with clinical potential, are also important directions for future research. Finally, given the inferential nature of MR, our findings should be interpreted as providing strong genetic evidence for potential causal links rather than as definitive proof of causation.

## CONCLUSION

In summary, this study systematically applied an MR framework to investigate the potential causal roles of the oral microbiota, blood metabolites, and inflammatory proteins in periodontitis risk. Our results provide robust genetic evidence, identifying family *Pasteurellaceae*, genus *Veillonella*, 45 blood metabolites, and 4 inflammatory proteins as causally associated with periodontitis susceptibility. Furthermore, preliminary mediation analysis suggests a potential causal pathway in which the protective effect of family *Pasteurellaceae* on periodontitis risk may be partly mediated by its influence on circulating 4-hydroxychlorothalonil levels. These findings offer insights into potential causal pathways beyond traditional observational associations, significantly deepening our understanding of the complex, multi-layered aetiology of periodontitis. The identified oral microbes, metabolites, and proteins are promising candidates for future risk assessment biomarkers and novel preventive and therapeutic targets for periodontitis. Furthermore, the identified mediation pathway provides a concrete hypothesis for future mechanistic studies aimed at dissecting the molecular dialogue between the oral microbiome and host metabolism in periodontal health.

### Acknowledgements

This study used GWAS data from prior research. Ethical approvals and consents were obtained in the original studies. The data and material that support the findings of this study are available in the GWAS Catalog (https://www.ebi.ac.uk/gwas/). We gratefully acknowledge the original authors for providing the data sets used in this research.

### Supplement Tables

**Table S1–S9**

https://www.quintessence-publishing.com/quintessenz/journals/articles/downloads/ohpd_2025_8030_zhao_tables_1-9.xlsx
